# Segmental Infantile Hemangiomas That Involve the Midline Define Risk for LUMBAR Syndrome

**DOI:** 10.1111/pde.70178

**Published:** 2026-04-23

**Authors:** D. Metry, E. Fernandez‐Faith, A. Haggstrom, K. Keppler‐Noreuil, I. J. Frieden

**Affiliations:** ^1^ Department of Dermatology Driscoll Children's Hospital, Texas A&M University Corpus Christi Texas USA; ^2^ Department of Dermatology Nationwide Children's Hospital, Ohio State University Columbus Ohio USA; ^3^ Department of Dermatology Indiana University Bloomington Indiana USA; ^4^ Department of Genetics University of Wisconsin Madison Wisconsin USA; ^5^ Department of Dermatology University of California San Francisco California USA; ^6^ Department of Pediatrics University of California San Francisco California USA

**Keywords:** LUMBAR syndrome, lumbosacral infantile hemangioma, segmental infantile hemangioma

## Abstract

A systematic review of clinical photographs from 91 patients with LUMBAR syndrome demonstrated that infantile hemangiomas in affected individuals were consistently segmental in morphology and involved the anatomic midline of the lumbosacral, sacrococcygeal, or pelvic regions. No cases were “partial segmental” or spared the midline. The hemangiomas were transmedian in all patients except one, in whom the hemangioma was unilateral and abutted the midline, and thus paramedian. These findings support the conclusion that segmental infantile hemangiomas involving the anatomic midline confer risk for LUMBAR syndrome and therefore warrant evaluation, while strictly lateral lesions without involvement of the midline do not. This refinement in risk stratification has immediate implications for clinical assessment and management.

## 1 Introduction

Diagnostic criteria for LUMBAR syndrome (lower body hemangioma, urogenital anomalies, spinal cord malformations, bony, anorectal, arterial and renal anomalies) were recently established through Delphi consensus. The syndrome is defined by a segmental infantile hemangioma (IH) of the lumbosacral, sacrococcygeal, or pelvic regions, accompanied by at least one other regional, characteristic congenital anomaly [1].

**FIGURE 1:**
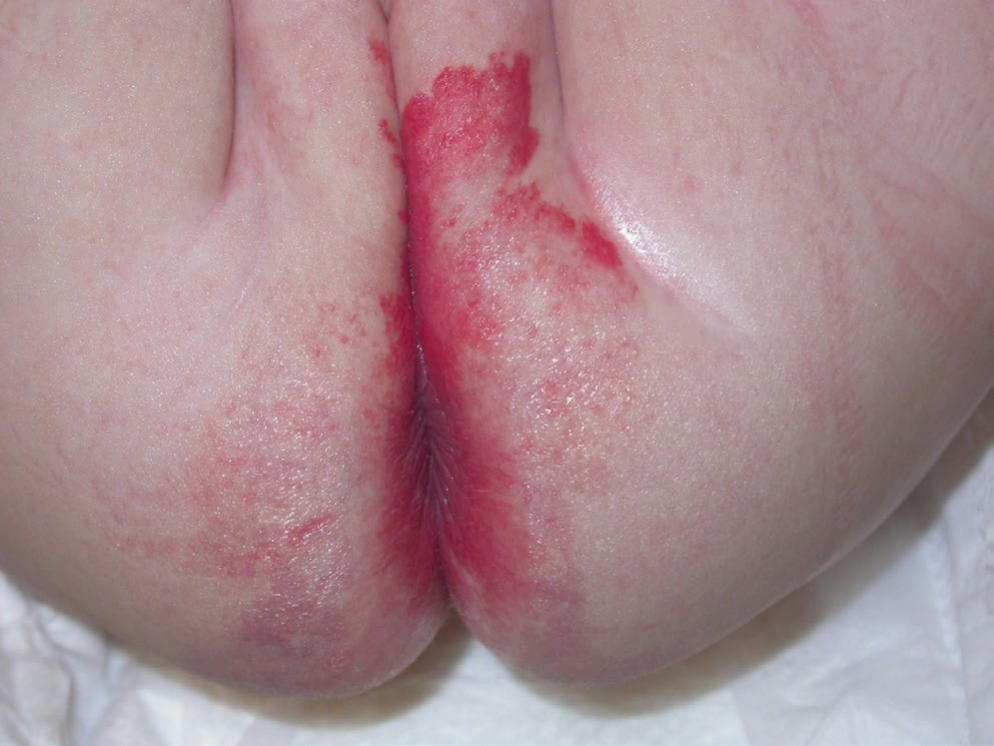
Infantile hemangioma with minimal or arrested growth (IH‐MAG) of the sacrococcygeal and pelvic regions that is segmental and transmedian, crossing the anatomic midline. This presentation warrants evaluation for LUMBAR syndrome.

**FIGURE 2:**
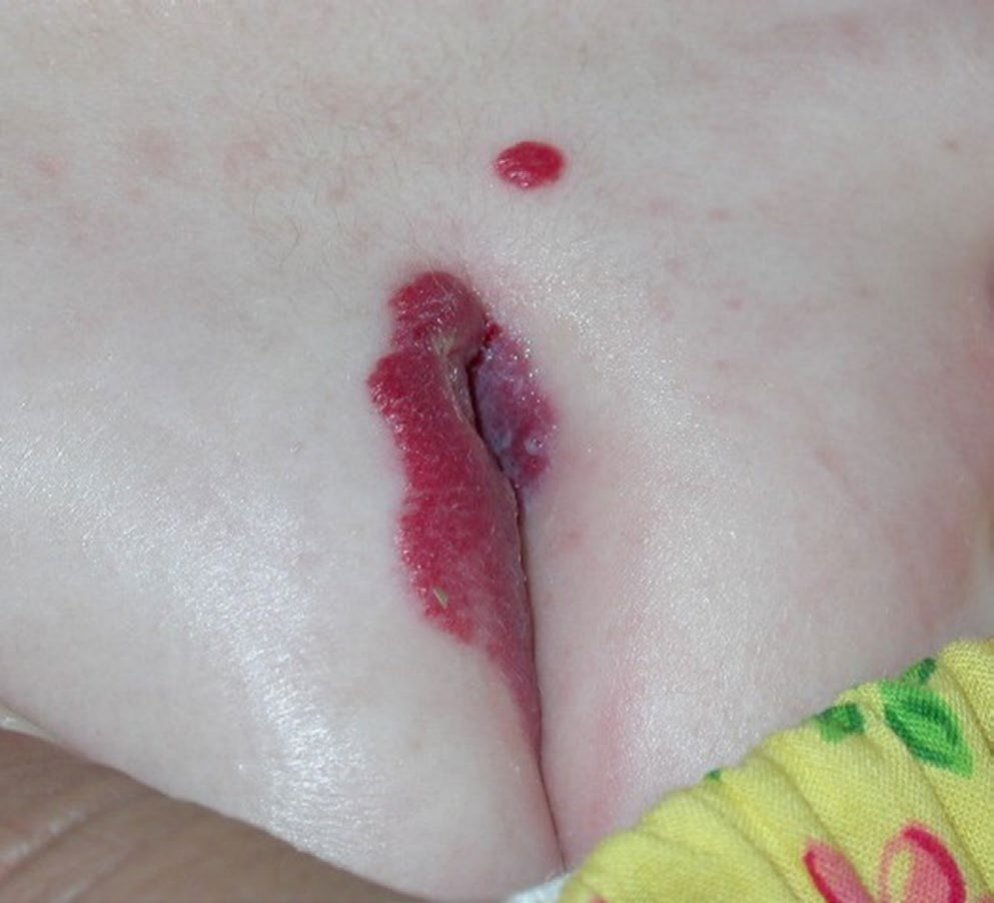
Infantile hemangioma of the sacrococcygeal region that is segmental and transmedian, crossing the anatomic midline. This presentation warrants evaluation for LUMBAR syndrome. A small localized infantile hemangioma is also present on the right within the lumbosacral region.

**FIGURE 3:**
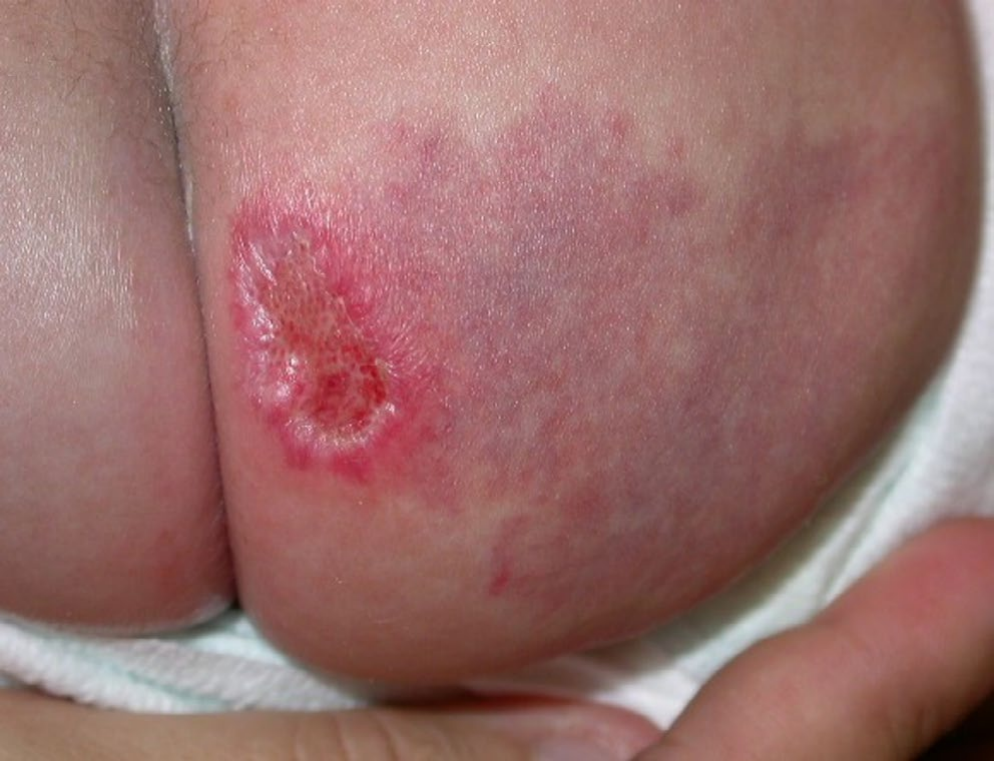
Ulcerated infantile hemangioma with minimal or arrested growth (IH‐MAG) of the right buttock that is segmental but strictly lateral, without involvement of the anatomic midline. In an otherwise healthy infant, this presentation does not warrant evaluation for LUMBAR syndrome.

**FIGURE 4:**
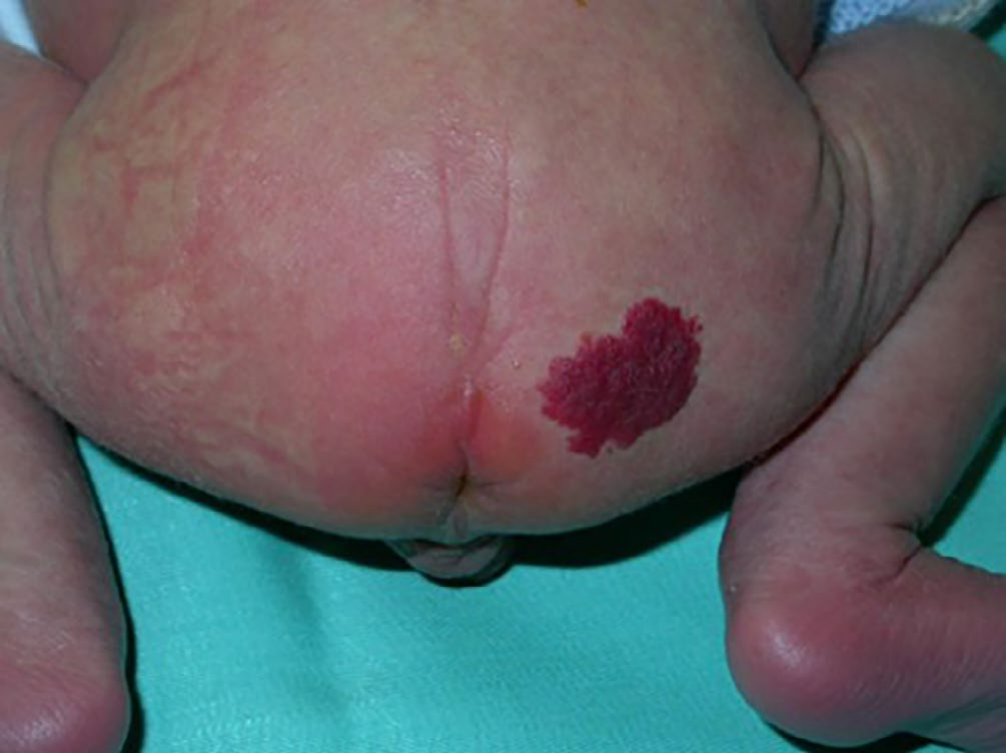
Infantile hemangioma of the right buttock that is segmental but strictly lateral, without involvement of the anatomic midline. In an otherwise healthy infant, this presentation does not warrant evaluation for LUMBAR syndrome. *Photograph courtesy of Dr. Eulalia Baselga*.

**FIGURE 5:**
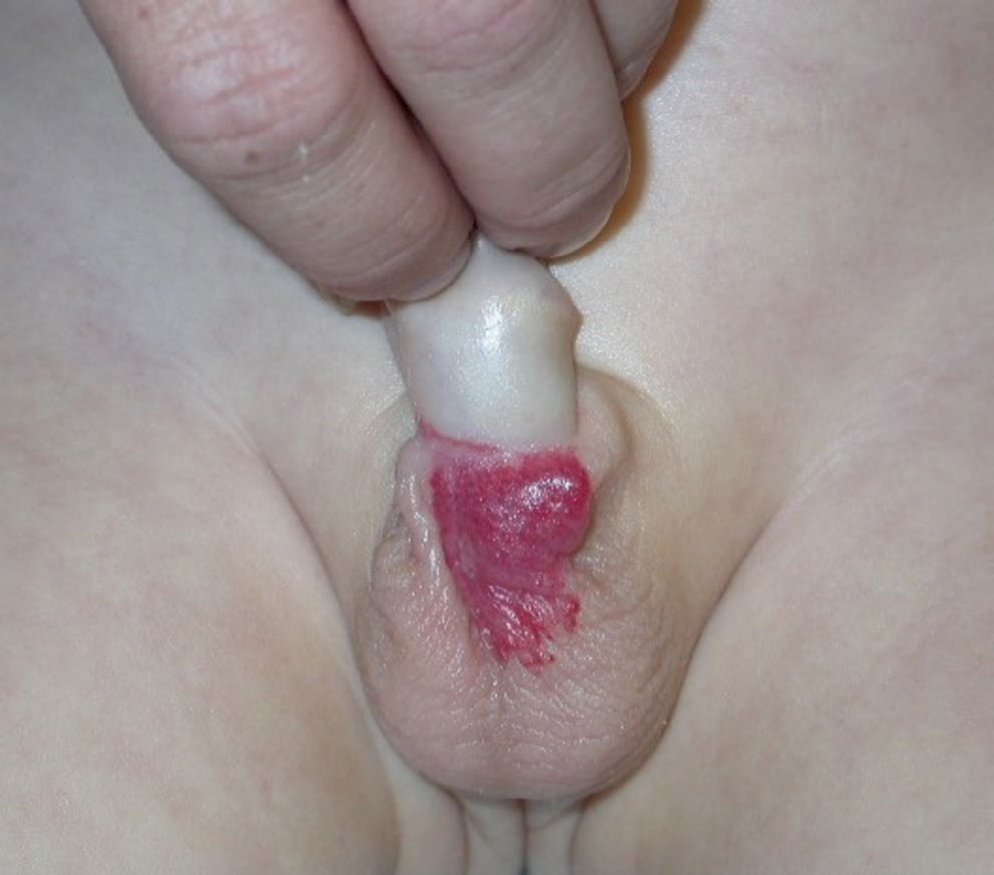
Infantile hemangioma of the scrotum that is unilateral and involves the anatomic midline but has *partial* segmental morphology. Although this lesion crosses slightly over the midline scrotal raphe, it still represents a unilateral, paramedian distribution. Based on our study, these unilateral and partial segmental features likely indicate a low risk for LUMBAR syndrome.

Segmental IH involve a broad territory of skin, rather than being spatially confined and/or appearing to arise from a central focus (Figures 1, 2, 3, 4). They are typically greater than or equal to 5 cm in diameter during infancy. While this size threshold is somewhat arbitrary, it has historical precedent for defining risk of PHACE syndrome. Unlike facial IH, non‐facial IH do not have well‐defined segmental maps. “Partial segmental” IH are smaller (usually 3–5 cm) and generally are not round or oval like “localized” IH (Figure 5). Originally described as “indeterminate”, subsequent mapping of facial segments has demonstrated that partial segmental IH represent portions of known embryonic facial segments. This principle likely also applies to IH occurring in the lumbosacral and other non‐facial regions. Partial segmental IH are thought to be low‐risk for IH syndromes, though their true risk is unknown [2]. Current guidelines recommend that all patients with segmental IH of the lumbosacral, sacrococcygeal, or pelvic regions undergo spinal, pelvic, and renal imaging to assess for LUMBAR syndrome [1, 3]. However, uncertainty remains regarding which IH pose the greatest risk and warrant evaluation.

Segmental infantile hemangiomas (IH) associated with LUMBAR frequently present as a distinct clinical subtype known as infantile hemangioma with minimal or arrested growth (IH‐MAG) [4]. (Figures 1 and 3) Unlike classic IH, IH‐MAG typically presents at birth or within the first few weeks of life as a flat, telangiectatic patch characterized by subtle erythema, superficial telangiectasias or venules, often admixed with or surrounded by areas of pallor. IH‐MAGs rarely undergo the rapid proliferative phase typical of classic IH; when proliferation does occur, it usually involves less than 25% of the lesion surface area. IH‐MAG exhibits a marked predilection for the lower body and is the predominant phenotype observed in LUMBAR, particularly in cases with limb involvement. Although gradual involution occurs over time, early complications, most notably ulceration, are common (Figure 3). Long‐term sequelae may include persistent soft tissue hypertrophy, lipoatrophy, and prominent superficial veins [4, 5].

## 2 Methods

We reviewed 148 cases of LUMBAR syndrome, including 141 previously published and 7 unpublished cases from the database used to establish the LUMBAR diagnostic criteria [1]. Of these, 91 patients (61.5%) had photographs adequate for confirming IH morphology and anatomic distribution. Each IH was assessed for morphology (segmental, partial segmental, localized) and whether lesions involved the anatomic midline. We used the terms “transmedian” for IH that crossed the anatomic midline and “paramedian” for those that abutted or “hugged” the midline but did not cross it. if Photographs of 4 cases with uncertain morphology were reviewed independently by the authors (D.M., E.F., A.H., I.F.) to establish majority consensus.

## 3 Results

All IHs were segmental; none were partial segmental or localized. Importantly, every case demonstrated midline involvement, regardless of subregion. Except for one case that was unilateral and paramedian [6], all IHs were transmedian, though often with marked asymmetry. No patient had a strictly lateral IH without midline involvement.

## 4 Discussion

Segmental IHs in LUMBAR syndrome likely involve the midline due to the embryologic origins of the overlying lumbosacral, perineal, and pelvic skin. These regions develop from the notochord, paraxial mesoderm, and cloacal membrane, which are central midline structures critical to the formation of the vertebral column, spinal cord, pelvis, and genitourinary tract [7, 8, 9]. Disruption of these midline developmental fields can result in both IH and congenital anomalies characteristic of LUMBAR syndrome.

In contrast, IHs in PHACE syndrome often occur in lateralized craniofacial segments, such as the temporal or auricular/periauricular regions. This reflects differences in embryogenesis, where craniofacial placodes and arterial territories follow neural crest migration patterns that need not cross the midline [2, 7, 10]. This distinction explains why PHACE‐associated IH may respect lateral boundaries, while LUMBAR‐associated lesions show strict midline involvement.

## 5 Conclusions

Our findings show that LUMBAR‐associated IH are invariably segmental, almost always transmedian, and rarely paramedian, but consistently involve the midline (Figures 1 and 2). In contrast, strictly lateral IH without midline involvement, and “partial segmental” IH appear unlikely to signal syndromic risk (Figures 3, 4, 5). Recognizing these distinctions can reduce unnecessary imaging and improve diagnostic accuracy. However, our study is limited by potential publication and selection bias and by the restricted availability of clinical photographs inherent to its retrospective design. Prospective studies are needed to validate these findings and to establish clear, evidence‐based screening guidelines for at‐risk infants.

## Consent

The authors have nothing to report.

## Conflicts of Interest

The authors declare no conflicts of interest.

## Data Availability

The data that support the findings of this study are available from the corresponding author upon reasonable request.
